# Evaluation of the Accurateness of the Nutritional Labels of Processed and Ultra-Processed Products Available in Supermarkets of Ecuador

**DOI:** 10.3390/nu12113481

**Published:** 2020-11-13

**Authors:** Diana Morales-Avilez, Carlos Cruz-Casarrubias, Lizbeth Tolentino-Mayo, Lorena Encalada-Torres, Victoria Abril-Ulloa

**Affiliations:** 1 Research Group Public Health, Food and Physical Activity in the Life Cycle, Medical Sciences Faculty, University of Cuenca, Av. 12 abril. Campus Paraíso, Cuenca 010104, Ecuador; diana.moralesa@ucuenca.edu.ec (D.M.-A.); lorena.encalada@ucuenca.edu.ec (L.E.-T.); 2 Center for Health and Nutrition Research, Mexican National Institute of Public Health Av. Universidad 655, Santa María Ahuacatitlán, 62100 Cuernavaca, Morelos, Mexico; casarrubiasnt@gmail.com (C.C.-C.); mltolentino@insp.mx (L.T.-M.); 3 El Colegio de Chihuahua, Partido Díaz 4723, Progresista, Ciudad Juárez, 32310 Chihuahua, Mexico

**Keywords:** nutrition label, traffic light label, ultra-processed products, sweeteners, obesity

## Abstract

Nutrition labeling is a public health tool that allows consumers to choose healthier foods and beverages. For this reason, there are protocols in place to monitor the food environment. The purpose of this study was to evaluate the consistency of nutrition labeling on packages for processed and ultra-processed products (UPPs) found at the main supermarkets in Cuenca, Ecuador. We conducted a descriptive cross-sectional study in which we identified label components on the packages of 1725 foods and beverages—including the nutrition table, statement on sweeteners, ingredient list, and the traffic light (TL) label—and determined the degree of consistency between the indicators reported on the traffic light label and those obtained from the information in the nutrition table. We found that 24% of products had inconsistencies in the traffic light label, and 10.9% of products had inconsistencies in the indicator for sugar specifically. The majority of inconsistencies were in the medium indicators for sugar (*K* = 0.68) and fat (0.75). The products with a medium indicator for sugar had a 1.98 (*p* < 0.05, 95% CI 1.15, 3.39) times higher chance of having inconsistencies in comparison with the low indicator. Health authorities must create new guidelines to allow for continued monitoring of the nutrition and traffic light labels on products accessible to consumers.

## 1. Introduction

According to data from the World Health Organization (WHO), the prevalence of obesity worldwide has continued to grow since 1975 [1]. In 2016, more than 650 million adults had obesity, 340 million children and adolescents were overweight or obese, and the number of preschoolers who were overweight reached 41 million [1]. According to a report on obesity worldwide, more than 70% of adults with overweight or obesity were from low- and middle-income countries [2]. In Ecuador, according to the results of the 2018 National Survey on Health and Nutrition in Ecuador (ENSANUT for its initials in Spanish), the prevalence of overweight and obesity combined has grown 5.5 percentage points (pp) in school children, 3.6 pp in adolescents, and 1.88 pp in adults relative to the 2012 ENSANUT [3,4].

With changes in the global food system, eating habits have shifted from a natural or minimally processed diet to greater consumption of ultra-processed products (UPPs) such as sugary beverages, soda, refined grains, baked goods, and sauces that are easy to prepare [5]. The latest report on UPPs (formulations of industrialized ingredients characterized as energy dense and/or high in nutrients of concern such as added sugars, saturated fat, and sodium) in Latin America (2015–2019) [6] suggests that the majority of countries in the region have reached a level of consumption of UPPs similar to what is observed in developed countries, where these products make up around 50% of dietary calories [7].

According to data published in ENSANUT—ECU 2012 about the consumption of processed and UPPs, of people surveyed nationwide from ages 10–19, in the seven days prior to the survey, 81.5% drank sugary beverages (soda, energy drinks, and processed juices with added sugar), 50.5% ate fast food (french fries, hamburgers, tacos, hot dogs, pizza, etc.), and 60% ate sweet or salty snack foods (cookies, french fries, chips, etc.). These trends could help explain the high prevalence of overweight among this population and could lead to a future rise in instances of type 2 diabetes and cardiovascular diseases—the main causes of mortality in Ecuador [4,8,9].

The obesity epidemic has prompted various international organizations, such as the Pan American Health Organization (PAHO), to generate action plans for preventing obesity in children and adolescents. Approved by member nations, the PAHO’s plan calls for better nutrition labeling on UPPs [5,7].

In order to decrease the prevalence of obesity and prevent non-communicable diseases (NCDs), governments must create and implement policies which reduce the consumption of UPPs and provide consumers with enough information so that they can identify and select foods with better nutritional quality [10].

Different countries worldwide, and in Latin America specifically, including Mexico, Peru, Chile, and Uruguay, have implemented front-of-package labeling on processed and UUPs [2]. Mexico had been using a system based on their dietary guidelines [11], but in March 2020, the country approved the warning label system used by Chile, Peru, and Uruguay [12].

In Ecuador, as part of the National Plan for Living Well (“Buen Vivir”) and based upon results from ENSANUT-ECU 2012, the government established a regulation for labeling on processed and UPPs, adopting a system of graphics which use traffic light colors. The traffic light label was implemented in 2014 as a strategy to address overweight and obesity by influencing dietary practices [13]. It should be noted that in Ecuador, qualitative studies have been carried out after the implementation of the traffic light (TL) system to find out the perceptions, knowledge, and practices of people when observing this graphic system, and the results indicate that the TL system has clear and precise information regarding the way to present the information about the fat, salt, and sugar content. The participants understood that the green and yellow colors reported in the nutritional traffic light are healthy for consumption, however, the red color represented an “alert”, but it did not mean to stop consuming the processed or UPPs [14], however, other studies in Ecuador reported that consumers consider that the price, flavor, and brand of the product are more important when selecting foods [15,16].

Ecuador has a federal regulation (RTE INEN 022 (2R) for its code) on “the labeling of processed and packaged foods products” [17] which establishes the requirements for the labeling of processed and UPPs to protect the health of consumers, in accordance with the principles of nutrition labeling established by the Codex Alimentarius Commission [18]. According to Ecuador’s regulation, processed and UPPs must include a traffic light (TL) label, with the TL consisting of three bars of the following colors: red means *high in*, yellow means *medium in*, and green means *low in*, placed horizontally on the food package, as indicated by the RTE INEN O22 (2R) regulation, in the upper left corner of the main panel or rear panel, therefore, the location of the TL will be at the discretion of the manufacturer, however, different studies indicate that frontal nutritional labeling systems allow consumers to choose healthier options [19,20,21]. These colors indicate the concentration of the components of sugars, fats, and sodium (salt) and are declared after a bromatological analysis [17]. The TL labeling in Ecuador is mandatory for all packaged processed and UPPs, and was developed according to the standards proposed by the UK Food Standards Agency in 2007 [22,23] and cut-off points were established according to PAHO recommendations [13]. The reporting of these critical nutrients in the packaging of processed and UPPs corresponds to policies implemented to inform the consumer about the nutritional content of the product and, with this information, create awareness to reduce their frequency of consumption and the health burden of problems related to nutrition such as cardiovascular diseases, diabetes, and cancer [24].

In addition to the TL label, there are other informative components present on the labels of processed and UPPs according to the International Network for Food and Obesity/Non-communicable Diseases (NCDs) Research, Monitoring and Action Support (INFORMAS) [25]—a global network which works to promote healthy food environments and reduce obesity and NCDs. The network has different modules for fulfilling its objective, one of which is “food labeling” [26]. Taking as reference the INFORMAS protocol for monitoring nutrition labels on processed and UPPs, as part of the surveillance of the food environment of industrialized products and taking into account the results of studies carried out in Spain and in the United States about irregularities in the nutritional labels with respect to the content of the product after a laboratory analysis, they found the under-declaration of the nutrient values on the labels [27,28], and they found higher or lower sugar values in relation to the values declared on the labels. In another study conducted in Canada [29], they found products with superior laboratory values compared to the values of calories, saturated fat, sugars, and sodium reported in the nutritional table. By focusing on the hypothesis that food labeling is a strategy that could help consumers make healthier choices, its success will depend on various aspects, such as the report of the nutritional TL indicators, and it is expected that their concordance will have minimal variability in relation to the expected indicators with the nutritional table, since this complementary information is useful for consumers when they select a processed and UPPs. The objective of this study was to evaluate the consistency of information reported on the nutrition labels of processed and UPPs found in the main supermarkets of Cuenca, a city in southern Ecuador.

## 2. Methods and Materials

This descriptive cross-sectional study involved collecting and analyzing information from photographs taken of processed and ultra-processed product labels in three main supermarkets in Cuenca. For each supermarket, permission was obtained to take photographs of processed and UPPs, and the study was designed to account for the greatest variety and availability of products by coordinating the schedules for data collection.

Three nutritionists took the photographs, having previously standardized their protocol with the method of analysis of nutrition label information proposed by INFORMAS [26].

Taking the photographs began in one supermarket, and the team photographed all available products in the store. The collection period lasted five days. In the two remaining supermarkets, photographs were taken of products that had not yet been registered, using a verification list to ensure no products were repeated. All photographs were reviewed, and in the instance that certain products had incomplete or low-quality photos, a team member(s) returned to the supermarket to photograph the item again.

Six photos were taken for each product, corresponding to each of the following components of the packaging: front of the package, barcode, nutrition table, traffic light label, ingredient list, and nutritional and health claims. To take the photographs, the team used the methodology explained by Kanter et al. [30], and coded each product by date, product category, and photograph number.

The devices used for taking the photographs included three cellphones, one cellphone with a dual camera, 48 MP (f/1.8, PDAF) + 5 MP (f/2.4, depth), dual, 12 MP (f/2.2, PDAF) + 5 MP (f/2.4, depth), and two cellphones with 12 MP camera, F/1.8 + f/2.4, phase detection autofocus, dual tone quad LED flash, HDR, optimal image stabilization, geo-tagging, touch focus, face recognition, panorama, 2160p @ 24/30/60 fps video, 1080p @ 30/60/120/240 fps, video light, 7 MP 1080p front camera @ 30 fps, f/2.2., HDR. Once the photographs of processed and UPPs were obtained, they were downloaded onto a computer and filed into separate folders by corresponding supermarket and product category. The data for the products were recorded in Excel spreadsheets. All data collection took place in September and October of 2019.

The collected information was classified into the following categories: sugar-sweetened beverages (carbonated drinks, savory drinks, nectars, and fruit juice with added sugar), breakfast cereals and granola, sausages, canned foods, cookies and crackers, fats and dressings (fat, sweet and savory sauces), dairy products (cheese, yogurt), bread and bakery products, sweet snacks (chocolate, flan, jellies, ice cream, pudding, and canned sweets), salty snacks, and other (chocolate powder, sweeteners, frozen fruit products, and fruit pulp). The study did not include candies or marshmallows.

### 2.1. Consistency of the Information Reported on the Nutrition Labels

In order to compare the quantities of nutrients reported on the nutrition table with those on the traffic light label, we used the Table 1 of components and permitted concentrations of fat, sugar, and salt from the Ecuadorian Technical Regulation INEN 022 (2R) included below [17]:

Nutrient information was obtained from the nutrition table and then standardized per 100 g and 100 mL. The contents of total fat and sugar were reported in grams, while the content of salt was reported in milligrams of sodium. We followed the instructions laid out in the federal regulation for generating the TL indicators; ice cream and yogurt was evaluated in milliliters and products which had to be prepared before consuming were evaluated according to the preparation instructions.

In order to identify products that contained non-caloric sweetener and had the statement “this product contains non-caloric sweetener” we used the ingredient listed and looked for sweeteners such as non-caloric artificial sweeteners (sucralose, acesulfame K, aspartame, neotame), natural sweeteners (stevia), and caloric sweeteners like polyalcohols (maltitol, mannitol, xylitol, isomaltitol, lactitol, insothiol, and erythritol). Inconsistencies were noted when the information reported on the nutrition label (statement on sweeteners and TL indicators) differed from what was observed on the ingredient list or obtained from the nutrition table.

### 2.2. Statistical Analysis

The data are reported by category and include frequencies, percentages, and 95% confidence intervals. Data analysis was conducted using the statistical package Stata version 14.0 and the kappa coefficient (*k)* was used to determine the degree of consistency between indicators on the TL label and the information obtained from the nutrition label. High consistency was considered for values of *k* > 0.8 according to a qualitative scale that rates concordance as follows: <0 less than chance agreement; 0.01–0.20 slight agreement; 0.21–0.40 fair agreement; 0.41–0.60 moderate agreement; 0.61–0.80 substantial agreement; 0.81–0.99 almost perfect agreement [31]. Three logistic regression models were also generated to determine the association between inconsistencies by nutrient indicator (sugar, fat, and salt) according to their content level (low, medium, and high). The three models included the indicator reported on the TL label and that obtained from the nutrition table. For the analysis of consistency in the TL indicators, 270 products were excluded because they did not have a TL label or because there were not required to according to the health regulations (for products without added sugar, fat, or salt and products that are exempt from nutrition labeling for having insignificant amounts of the required nutrients) [17,32]. Statistical significance was considered to be a *p* value < 0.05.

## 3. Results

Out of the total of 1725 processed and UPPs, 15.4% were beverages, 12.2% fats and dressings, 12% cookies and crackers, 10.4% canned foods, 10% sweet snacks, 9.3% bread and bakery products, 7.9% breakfast cereals and granola, 7.7% dairy products (cheeses and yogurt), 7.3% salty snacks, 4.4% other (chocolate powder, sweeteners, frozen fruit products, and fruit pulp), and 3.3% sausages. Our analysis found that 14.3% of these products had the statement “contains non-caloric sweetener,” 93.4% displayed the traffic light nutrition label, 99.3% reported the ingredient list, and 100% displayed the nutrition table (Table 2).

Table 3 shows the results of the indicators reported in the TL and the indicators expected. According to the sugar indicator, there was a higher proportion of sugar-sweetened beverages (49.0%) and sweet snacks (9.0%) classified as “medium” compared to the expected indicator for these categories (41.6% and 3.9%, respectively; these differences were reversed for the “high” indicator for sugar-sweetened beverages (27.4% expected vs. 17.1% reported) and sweet snacks (93.6% expected vs. 88.4% reported).

Table 4 shows the results of inconsistencies found between components of the nutrition label. We found that products which did not have a TL label but were required to do so included beverages, dairy products (cheeses, yogurt), bread and bakery products, and sweet snacks. Products that contained sweeteners but did not have the statement “contains non-caloric sweetener” included beverages (4.9%), breakfast cereals and granola (6.6%), cookies and crackers (3.4%), fats and dressings (1.4%), bread and bakery products (3.8%), sweet snacks (3.5%), salty snacks (0.8%), and other (chocolate powder, sweeteners, frozen fruit products, and fruit pulp) (5.3%). In general, 27.1% of the products had inconsistencies in the TL indicators, primarily bread and bakery products (37.5%) and dairy products (34.3%). Among all the food categories, there was a higher instance of inconsistency for the TL indicator for sugar (10.9%) in comparison to the indicators for fat (10.0%) and salt (8.8%).

**Figure 1:**
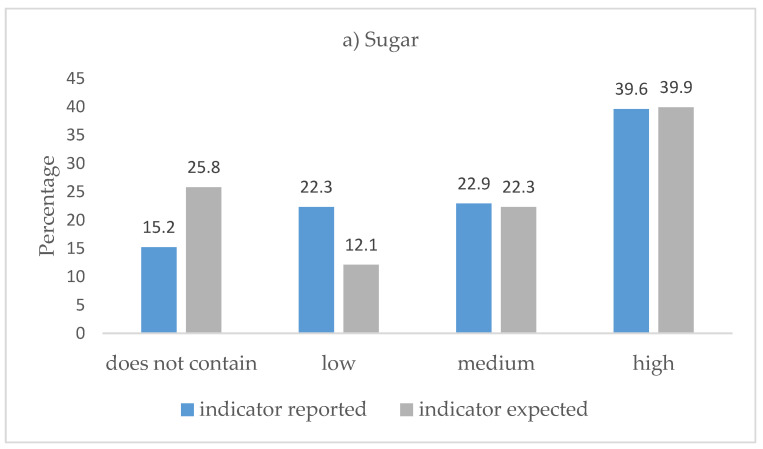

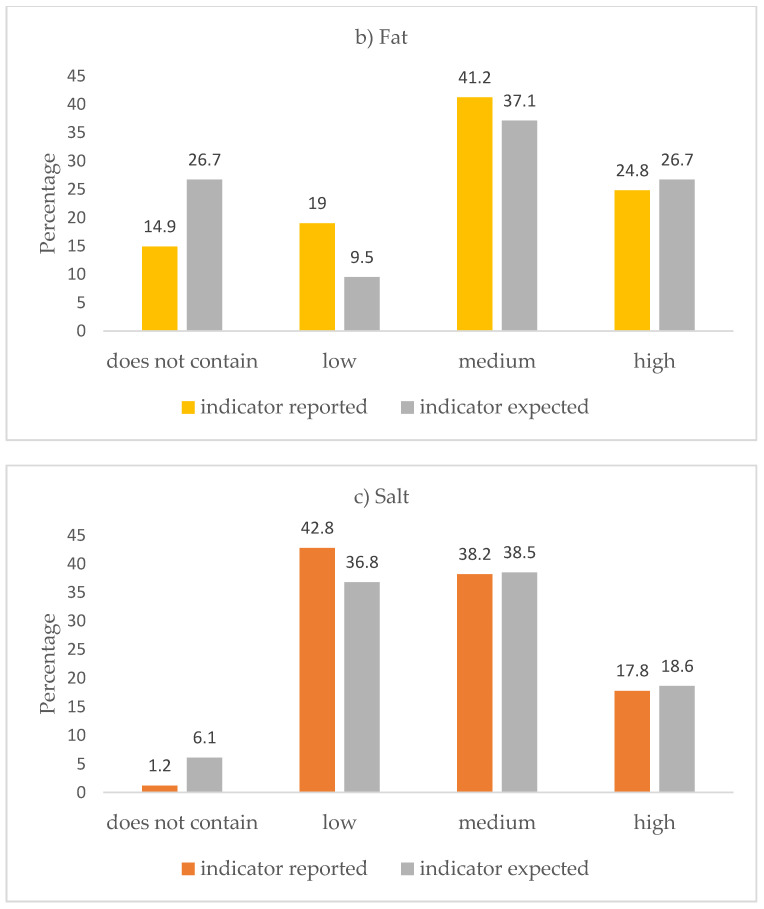
Proportion of TL indicators reported and expected by type of nutrient (n = 1725). The indicator “does not contain” was considered when the values of sugar, fat, or sodium were zero; (**a**) chart for sugar, (**b**) chart for fat, (**c**) chart for salt (sodium).

The proportion of indicator levels reported on the analyzed labels and their expected proportions are shown in Figure 1. For sugar and fat, the proportion of products with the “high” indicator is less than what was expected (24.8% vs. 26.7%). This difference is reversed for the “low” indicator, where for all three nutrients, the proportion of labels marked “low” was higher than the estimate.

In the indicators for sugar, there was good consistency between the values reported on the nutrition table and the concentration indicators reported on the TL label, 93.6% (*K* = 0.88) of products with the “low” indicator and 90.8% (*K* = 0.84) of the products with a “high” indicator. The majority of inconsistencies were found in the “medium” indicator (*K* = 0.68) and it was most often that levels should have been considered “high” (15.0%) according to the expected indicator. The inconsistencies for the indicator “medium” for fat were higher (*K* = 0.75) compared to the indicators “low” (*K* = 0.86) and “high” (*K* = 0.86); 8.5% of the analyzed products should have been reported in the “high” category. For salt, there was an adequate consistency for the low (92.5%), medium (90.5%), and high (90.5%) concentrations (Table 5).

Table 6 shows the odds of having inconsistencies in the reported TL indicators, which are highest for the “medium” indicator in fat (OR = 3.95, *p* < 0.001, CI 95% 2.42, 6.45) and sugar (OR = 1.98, *p* < 0.05, IC 95% 1.15, 3.39) compared to the “low” indicator. For the expected indicators, products classified as “high” in salt had a 4.16 (*p* < 0.001, IC 95% 4.96, 8.42) times higher probability of having inconsistencies in comparison to the low indicator.

## 4. Discussion

The results of this study demonstrate that nutrition labels for processed and UPPs in Ecuador have inconsistencies in the reported concentrations on the traffic light label, primarily for sugar content in that the values reported are lower than what should be used. Among the nutrition-related labeling components that were identified, we found the statement “contains non-caloric sweetener” in all of the food categories except the sausages, canned foods, and salty snacks. This finding is interesting to highlight because it suggests that the majority of sweet-tasting industrialized products contain sweeteners. Furthermore, a study in Mexico that evaluated the nutritional quality of sugar-sweetened beverages reported that 48.2% contained sweeteners [33]; in our results, we found that this proportion was higher (approximately 58%). These data allow us to know that consumers in Ecuador are more exposed to these additives in beverages. However, it is necessary to modify the regulations to declare the quantity of these. In two reviews about the use of sweeteners, the results are controversial since some studies have found cytotoxic and metabolic effects, while others have not. Although recognizing that one benefit of sweeteners might be weight loss, the reviews’ authors still emphasize the need for longitudinal studies over adequate amounts of time to determine the effects of sweeteners on human health [34,35]. The consumption of sweeteners, commonly found in UPPs, promotes habituation to the sweet taste and this is associated with a higher intake of products with excessive amounts of sugar and, for this reason, they are not recommended for children because of the eating habits that are acquired at an early age [22].

Among the other nutritional components of food packaging considered in this study, we found that 93.4% of products displayed the TL label, 93.3% displayed the ingredient list, and 100% reported the nutrition table. The reason not all 100% of products had the TL label could be because of certain exceptions described in the Nutrition Labeling of the Ecuadorian Technical Standard (NTE in Spanish) document INEN 1334-2 [32] or because certain processed products may not have had added fat, sugar, or salt [17].

In this study, differences were observed in the level of the indicators for each nutrient. It was more frequent for some food categories to report an indicator as “medium” when they should be reported as “high”, especially in sugar-sweetened beverages for the sugar indicator. In a study carried out in Spain about the sugar content in processed products, they found that in the 28 categories of products analyzed, 10 had high sugar content in relation to the tolerance levels of the European Union [27]. In the United States studies, they found that 19% of the products in the sugar component presented lower data than their analytical data [28]. In the Canadian study, they found that of the 1010 products evaluated, 16.7% contained an excess of nutrients in relation to the information in the nutritional table, specifically 15.8% for saturated fats, 14.2% for calories, and 12.9% for sugars [29]. These results demonstrate the importance of monitoring nutritional labels in order to take actions that benefit the health of consumers.

In terms of inconsistencies in the nutrition label, 2.8% of processed and UPPs that did not have the statement “this product contains non-caloric sweetener” still had the name of a sweetener in the ingredient list. According to the regulation RTE INEN 022 (2R), this statement is required if there are one or more non-caloric sweeteners in the ingredient list. Regarding the category of dairy products that represents the highest percentage of inconsistencies with respect to sugar content (22.6%), these results may be in relation to the definition established in the RTE INEN 022 on sugars: “Monosaccharides and disaccharides are understood present in processed foods from all sources, whether own or added” [17]. This allows us to understand that the nutritional table “sugars” includes intrinsic sugars and does not specifically detail the amount of added sugars. This could be revised for government agencies that regulate labeling regulations in Ecuador to give more effective and adequate messages to the population and, in addition, guarantee a satisfactory report in the TL indicators because the information reported in these is more reliable and easier to understand according to a study that evaluated the perceptions of consumers about the formats of the front labels in 12 countries [36].

Regarding the phrase “does not contain,” according to RTE INEN 022 (2R), this phrase should be used on the TL label when a processed products does not contain one of the three components— sugar, fat, or salt [17]. That being said, as seen in Figure 1, our study found that “does not contain” should have been reported in greater proportion for all three components of the TL. However, the “low” category is defined by RTE INEN 022 (2R) as a “< than” cut-off point, widening the range of what can be classified as “low” and allowing companies to show industrialized products as “low in”, which has a greater impact on consumer preferences through the positive signal of green coloring. Indeed, in a study conducted in Peru on front-of-package label design preferences between the octagon warning system and traffic light–GDA (Guideline Daily Amount) system, participants favored the traffic light system and one of the reasons was that “it has more green, so it is more healthy” [37].

As for the proportion of products that should have had a “high” concentration of sugar and fat on the TL label, the reported indicators were underestimated. However, the proportion of “low” indicators reported was overestimated for all three nutrient components. These results about the levels of sugar and fat are important. A study from Quito, Ecuador, published in 2017, found that consumers paid more attention to these two components, sugar and fat, while grocery shopping in order to maintain health, prevent disease, and stay informed [38]. The results of our study, however, suggest that consumers are not necessarily receiving reliable information from the TL label.

In terms of evaluating concentration levels for the TL component, our results show that the majority of inconsistencies are in the “medium” concentration for the sugar component; 15.0% of the products analyzed should have been reported as “high” in sugar but were reported as “medium.” A study on the TL label that took place in Ecuador found that 13% of products had inconsistencies in the salt component, since values would be overestimated when comparing the observed to expected colors in the graphic TL system. The authors suggested these inconsistencies might possibly be attributed to the calculator for food labeling available on the website for the National Agency for Health Regulation, Control, and Surveillance (ARCSA) [39,40]. However, this does not justify the inconsistencies because the food industry is able to conduct a simple analysis to calculate and determine the correct TL level for their products.

In the case of Ecuador, the RTE INEN 022 (2R) regulation includes the sanctions regime section that is supported in Law No. 2007-76 of the Ecuadorian Quality System. This law indicates sanctions to producers, importers, or suppliers of goods and services subject to technical regulations that fail to comply with the presentation of the certificate of conformity [41]; this is obtained when a product or service meets the requirements of the technical regulation. Although these sanctions can be fines ranging from one thousand to ten thousand dollars for different infractions, however, in the RTE INEN 022 (2R) regulation, there is no specific section that details the penalties by type of infraction in the requirements that must comply with nutritional labeling, one of them being the inconsistency in the content of the components of the nutritional traffic light. On the other hand, some countries worldwide have implemented warning front labels [42], however, when reviewing the regulations or standards for labeling food and non-alcoholic beverages, countries such as Mexico [43], Chile [44], and Peru [45] do not report any regulation with specific sanctions in the case of presenting inconsistencies in the frontal labeling, which, without due vigilance, harms consumers if they select a product that does not really contain the amount of critical nutrients that the label reports; this could increase the prevalence of NCDs and have an impact on the family economy and health systems.

Compared to the results of previous studies, the data found here are important because they evaluate the nutritional quality of products based on what is reported on their packaging, while previous studies focused on the nutrition label have used qualitative methods to explore consumer perception around the implementation of the TL label. In Ecuador, there is still no study that relates nutritional status with the consumption of processed and UPPs after implementing the TL, however, the literature indicates that one should wait around 5 years after a policy is initiated to see this type of change and this may be one of the reasons why there are still no published studies [46]. According to PAHO reports on processed products and their sales per capita, the first report that gathered information from 2000 to 2013 includes data from Ecuador, but at that time the nutritional traffic light was not yet implemented. Then, in the second PAHO report (2009–2014), there are no figures for Ecuador, only for seven countries: Argentina, Brazil, Chile, Colombia, Mexico, Peru, and Venezuela. In a study by Freire et al. in 2015 [14], involving children and adolescents, the results suggested that the presence of the TL label is often unimportant when selecting a product because flavor, brand name, and available money are all more relevant factors. In a study by Padilla et al. in 2016 [15], a group of people ages 18–40 did not feel they had stopped consuming certain products after having read the contents of the TL label. In another study carried out by Guevara in 2015 [16], the participants indicated that they purchase products by looking at the price before considering other characteristics, and that food selection had to match the preferences of their children to avoid wasting food and money. Nonetheless, we believe it is important to evaluate how the food industry is complying with the provisions of the Ecuadorian Technical Regulation INEN 022 which was created to protect the health of the Ecuadorian population and was based upon the epidemiological profile on morbidity.

In a study from New Zealand which evaluated the nutrition label, researchers found 156 processed and UPPs that were eligible for a health claim despite having at least one red bar on their traffic light labels. In accordance with the Ecuadorian Technical Standard on nutrition and health claims, it would be important to analyze products which have health-related messages in relation to their TL indicators [47,48].

Within Latin America, Chile and Mexico have conducted research on food labeling using the protocol from INFORMAS [26,49]. In Chile, a research group developed a validated method for analyzing nutrition labels using photography. The study consisted of taking photographs in 11 supermarkets to evaluate and monitor the food environment. The researchers obtained photographs of 10,000 products and the information on the food labels was registered in a database. This information has served to guide and evaluate actions within the context of the obesity and non-communicable disease epidemics [30].

In Chile, following the implementation of the initial Food Labeling and Advertising Law, there was a significant reduction in the number of products containing large amounts of sugar and sodium [50]. In Ecuador, one part of a study by Peñaherrera et al. focused on changes in the amount of sugar in soft drinks before and after the implementation of the TL label. The results from after the implementation indicate that of 14 different soda types, seven had a lower sugar content, four had a higher sugar content, and three showed no difference in content [51]. Although our study here does not analyze changes before and after the TL label, the inconsistencies found in the “medium” level indicators for fat and sugar can help public health decision-makers to develop guidelines for better surveillance and control of the TL label so that consumers will have an accurate understanding of the nutritional content of products as they make purchasing decisions.

This is the first study in Ecuador to analyze the consistency of the nutrition label on processed and UPP packages using the methodology from the nutrition labeling module of the INFORMAS network and Ecuadorian Technical Regulation (2R), obtaining results which will inform future actions to improve the nutrition label so that consumers can make healthier food choices. In addition, this study will be able to report its findings about the nutrition label in Ecuador to the international INFORMAS network.

In this study, one of the limitations was that the majority of the packages for candies and marshmallows were transparent and so the information in a photograph could not be read clearly. Therefore, the study did not include these products and instead prioritized taking photographs of the other food categories, taking into account the amount of time allowed by each supermarket to carry out the research activity.

Among other limitations, this study described the consistency of the TL nutrition label on processed and UPPs, but other studies are needed to understand consumer behavior when reading the nutrition label in order to know which products are purchased and consumed most often.

## 5. Conclusions

The results showed inconsistencies in the fat, sugar, and salt indicators between the expected values and the values reported in the TL of the processed and ultra-processed products. Therefore, it is essential that the technical bodies in charge of the control and sanitary surveillance of these products exercise new and strong nutritional information policies so that the manufacturers comply with the provisions of the laws and regulations implemented in the countries on the regulation of the nutritional labeling or otherwise the respective sanction is exercised on them in case of non-compliance, because if the labeling wrongly informs the content of critical nutrients, this information will be useless to prevent the epidemic of overweight and obesity, as well as to reduce the prevalence of chronic non-communicable diseases. Moreover, this could affect the health of consumers. In addition, it is also essential to reconsider and update the system of graphics used on these products, which, added to the real information of the critical nutrients in it, will guarantee effective consumer protection, allowing the selection of foods with an adequate report on nutritional quantity and quality.

## Figures and Tables

**Table 1:** Components and permitted concentrations.

Components	Level		
	“Low” Concentration	“Medium” Concentration	“High” Concentration
Total Fat	≤3 g per 100 g	>3 g and <20 g per 100 g	≥20 g per 100 g
	≤1.5 g per 100 mL	>1.5 g and <10 g per 100 mL	≥10 g per 100 mL
Sugar	≤5 g per 100 g	>5 g and <15 g per 100 g	≥15 g per 100 g
	≤2.5 g per 100 mL	>2.5 g and <7.5 g per 100 mL	≥7.5 g per 100 mL
Salt (Sodium)	≤120 mg per 100 g	>120 mg and <600 mg per 100 g	≥600 mg per 100 g
	≤120 mg per 100 mL	>120 mg and <600 mg per 100 mL	≥600 mg per 100 mL

Source: Ecuadorian Technical Regulation INEN 022 (2R) [17].

**Table 2:** Proportion of nutrition components found on the product label by category of food.

	Total Products n %		Statement on Sweeteners %	Traffic Light Label %	Ingredient List %	Nutrition Table %
Sugar-sweetened beverages	265	15.4	58.1	94.3	100.0	100.0
Breakfast cereals and granola	137	7.9	5.8	89.8	100.0	100.0
Sausages	61	3.5	0.0	100.0	100.0	100.0
Canned foods	179	10.4	0.0	100.0	100.0	100.0
Cookies and crackers	207	12.0	5.8	100.0	99.5	100.0
Fats and dressings	210	12.2	1.4	93.3	100.0	100.0
Dairy products	133	7.7	15.8	91.0	100.0	100.0
Bread and bakery products	160	9.3	4.4	97.5	100.0	100.0
Sweet snacks	173	10.0	16.2	94.2	100.0	100.0
Salty snacks	125	7.3	0.0	98.4	97.6	100.0
Other	75	4.4	17.3	42.7	88.0	100.0
Total	1725	100.0	14.3	93.4	99.3	100.0

Note: The classification of processed products was done out of convenience while trying to conserve some categories which would be comparable to other food classification frameworks.

**Table 3:** Proportion of TL indicators reported on the label and indicators expected by product category.

	Low		Medium		High	
	Indicator Reported %	Indicator Expected %	Indicator Reported %	Indicator Expected %	Indicator Reported %	Indicator Expected %
**Sugar indicator**						
Sugar-sweetened beverages	33.9	31.0	49.0	41.6	17.1	27.4
Breakfast cereals and granola	11.6	10.7	27.3	30.6	61.2	58.7
Sausages	100.0	100.0	0.0	0.0	0.0	0.0
Canned foods	97.5	97.5	2.5	1.3	0.0	1.3
Cookies and crackers	11.4	16.3	19.3	16.8	69.3	66.8
Fats and dressings	40.4	39.8	19.3	24.6	40.4	35.7
Dairy products	41.2	42.2	23.5	24.5	35.3	33.3
Bread and bakery products	30.2	33.8	37.5	35.3	32.4	30.9
Sweet snacks	2.6	2.6	9.0	3.9	88.4	93.6
Salty snacks	87.4	85.4	7.8	9.7	4.9	4.9
Other	43.5	43.5	26.1	4.4	30.4	52.2
Total	38.1	38.4	23.2	21.5	38.7	40.1
**Total fat indicator**						
Sugar-sweetened beverages	77.6	78.8	22.0	20.8	0.4	0.4
Breakfast cereals and granola	33.1	29.8	63.6	62.0	3.3	8.3
Sausages	6.7	6.7	80.0	86.7	13.3	6.7
Canned foods	44.9	41.8	48.1	50.6	7.0	7.6
Cookies and crackers	0.0	0.5	55.5	50.0	44.6	49.5
Fats and dressings	52.6	56.7	19.3	15.2	28.1	28.1
Dairy products	13.7	24.5	63.7	46.1	22.6	29.4
Bread and bakery products	14.0	19.1	70.6	66.9	15.4	14.0
Sweet snacks	38.1	39.4	14.2	14.2	47.7	46.5
Salty snacks	0.0	0.0	20.4	12.6	79.6	87.4
Other	39.1	43.5	56.5	47.8	4.4	8.7
Total	34.5	36.1	40.6	37.0	25.0	26.9
**Salt indicator**						
Sugar-sweetened beverages	99.2	97.6	0.8	2.5	0.0	0.0
Breakfast cereals and granola	43.8	44.6	52.9	52.1	3.3	3.3
Sausages	0.0	0.0	0.0	13.3	100.0	86.7
Canned foods	6.3	3.8	63.9	66.5	29.8	29.8
Cookies and crackers	19.8	14.9	65.8	65.4	14.4	19.8
Fats and dressings	14.6	17.0	26.3	24.6	59.1	58.5
Dairy products	65.7	62.8	23.5	25.5	10.8	11.8
Bread and bakery products	14.0	14.7	75.7	71.3	10.3	14.0
Sweet snacks	93.6	87.7	6.5	12.3	0.0	0.0
Salty snacks	11.7	13.6	53.4	54.4	35.0	32.0
Other	52.2	43.5	39.1	47.8	8.7	8.7
Total	43.8	42.1	38.2	39.1	18.1	18.9

Note: Expected indicator was obtained with the information from the nutritional table of the packaging of each processed and ultra-processed product (UPP).

**Table 4:** Proportion of products with inconsistencies in the traffic light label and statement on sweeteners by category of food.

					Inconsistency in the TL Indicator			
	Total n %		TL Not Shown * %	Statement on Sweeteners %	TL Indicators ** %	Sugar %	Fat %	Salt %
Sugar-sweetened beverages	265	21.1	0.4	4.9	17.6	13.9	1.6	1.5
Breakfast cereals and granola	137	24.8	0.0	6.6	23.1	5.0	7.4	6.6
Sausages	61	36.1	0.0	0.0	20.0	0.0	13.3	23.0
Canned foods	179	15.1	0.0	0.0	14.6	1.9	6.3	7.3
Cookies and crackers	207	28.5	0.0	3.4	24.8	8.9	10.9	12.1
Fats and dressings	210	32.9	0.5	1.4	31.6	17.5	9.9	10.0
Dairy products	133	30.1	1.5	0.0	34.3	22.6	3.9	3.8
Bread and bakery products	160	46.9	1.3	3.8	37.5	16.9	10.3	11.3
Sweet snacks	173	21.4	0.6	3.5	15.5	6.5	9.7	9.8
Salty snacks	125	24.0	0.0	0.8	17.5	3.9	8.7	13.6
Other	75	25.3	0.0	5.3	60.9	21.7	34.8	10.7
Total	1725	27.1	0.4	2.8	24.0	10.9	10.0	8.8

Note: The data represent the proportion of products with inconsistencies by category of food. * Proportion of products that do not display a traffic light (TL) label and that should according to health regulations. ** Proportion of products that have inconsistencies in any TL indicators of those that have the TL label and that should according to regulation.

**Table 5:** Consistency between the information reported on the TL label and the estimation based on the nutrition table (n = 1455).

		Calculated Indicator			
		Low	Medium	High	Consistency
**Reported Indicator**	n	%	%	%	*K*
Sugar					
**Low**	545	93.6	5.1	1.3	0.8888
**Medium**	334	10.5	74.6	15.0	0.6858
**High**	576	1.0	8.2	90.8	0.8421
Fat					
**Low**	494	93.3	6.5	0.2	0.8599
**Medium**	599	10.7	80.8	8.5	0.7537
**High**	362	0.6	6.6	92.8	0.8599
Salt					
**Low**	640	92.5	6.6	0.9	0.8867
**Medium**	556	4.7	90.5	4.9	0.8401
**High**	259	2.7	5.8	91.5	0.8729

K: kappa coefficient, values above 0.8 indicate good consistency.

**Table 6:** Odds ratio for having inconsistencies by type of TL indicator reported on the label (n = 1455).

	Sugar		Fat		Salt	
Reported indicator	OR	CI 95%	OR	CI 95%	OR	CI 95%
**Low**	1.0	-	1.0	-	1.0	-
**Medium**	1.98 *	[1.15, 3.39]	3.95 ***	[2.42, 6.45]	0.50 *	[0.29, 0.85]
**High**	1.23	[0.65, 2.35]	0.71	[0.34, 1.50]	0.31 **	[0.14, 0.68]
Expected indicator						
**Low**	1.0	-	1.0	-	1.0	-
**Medium**	1.05	[0.61, 1.81]	0.28 ***	[1.72, 0.45]	1.92 *	[1.12, 3.28]
**High**	0.91	[0.48, 1.72]	0.94	[0.48, 1.87]	4.16 ***	[1.96, 8.42]

Adjusted for the expected indicators, in all models, the “low” category was considered as a reference. * (*p* < 0.05), ** (*p* < 0.01), *** (*p* < 0.001) indicate level of significance.
